# *BMC Microbiology* reviewer acknowledgement 2015

**DOI:** 10.1186/s12866-016-0641-7

**Published:** 2016-03-01

**Authors:** Hilary Logan

**Affiliations:** BioMed Central, Floor 6, 236 Gray’s Inn Road, London, WC1X 8HB UK

## Abstract

The editors of *BMC Microbiology* would like to thank all our reviewers who have contributed to the journal in Volume 15 (2015).

**Mohamed Abdel-Maksoud**

Egypt

**Robert Abramovitch**

USA

**Jacqueline Abranches**

USA

**Abu-Bakr Abu-Median**

UK

**La'Tonzia Adams**

USA

**Rasheed Adeleke**

South Africa

**Tomabu Adjobimey**

Germany

**Ameeta Agarwal**

USA

**Ma Guadalupe Aguilera-Arreola**

Mexico

**Jesus Aguirre**

Mexico

**Amir Aharoni**

Israel

**Warish Ahmed**

Australia

**Juhee Ahn**

South Korea

**Manuel Aira**

Spain

**Dragana Ajdic**

USA

**Thomas Åkerlund**

Sweden

**Masato Akiba**

Japan

**Ezekiel O Akinkunmi**

Nigeria

**Pedro Alcolea**

Spain

**Herve Alexandre**

France

**Syed Abid Ali**

Pakistan

**Robert Allaker**

UK

**Nerino Allocati**

Italy

**Qadreyah Al-Matawah**

Kuwait

**Fausto Almeida**

Brazil

**Andrew Alspaugh**

USA

**Emrah Altindis**

USA

**Sergio Alvarez-Perez**

Spain

**Artur Alves**

Portugal

**Carlos Amabile-Cuevas**

Mexico

**Nana Ama Amissah**

Ghana

**Hany Anany**

Canada

**Hisashi Anbutsu**

Japan

**Annette Anderson**

Germany

**Jonathan Anderson**

Australia

**Anna Paola Andolfo**

Italy

**Bruno Andrade**

Brazil

**Genevieve Andre-Fontaine**

France

**Simon Andrews**

UK

**Letizia Angiolella**

Italy

**Kingsley Chidozie Anukam**

Nigeria

**Charles Apperson**

USA

**Victor Aquino**

Brazil

**Virginia Aragon**

Spain

**Jesús Aranda**

Spain

**Demelash Areda**

USA

**Jesús Arenas**

The Netherlands

**Hector Arguello Rodriguez**

Spain

**Agustin Ariño**

Spain

**Stefania Arioli**

Italy

**Guillaume Arlet**

France

**Pankaj Arora**

South Korea

**Corinne Arpin**

France

**Mikko Arvas**

Finland

**Jonathon Audia**

USA

**Victoria Auerbuch**

USA

**Swinburne Augustine**

USA

**Ruben Avendaño-Herrera**

Chile

**Stéphane Aymerich**

France

**Abdu Azad**

USA

**Taj Azarian**

USA

**Rosa Aznar**

Spain

**Paul Babitzke**

USA

**Herwig Bachmann**

The Netherlands

**Prasanta Bag**

India

**Tamishraha Bagchi**

India

**Martin Bahl**

Denmark

**Linquan Bai**

China

**Michael Bailey**

USA

**Matthew Bakker**

USA

**Guus Bakkeren**

Canada

**Manju Bala**

India

**Regina Baldini**

Brazil

**Peter Balint-Kurti**

USA

**Agim Ballvora**

Germany

**David Baltrus**

USA

**Stefania Bambini**

Italy

**Agata Bancerz-Kisiel**

Poland

**Debdulal Banerjee**

India

**Yiming Bao**

USA

**Jeri Barak**

USA

**Olivia Bargiacchi**

Italy

**Debmalya Barh**

India

**Emma Barnard**

USA

**Joao Bassin**

Brazil

**Joyoti Basu**

India

**Sulagna Basu**

India

**Margaret Bauer**

USA

**Anthony Baughn**

USA

**Alexis Bazire**

France

**Michael Beeton**

UK

**Alexander Beliaev**

USA

**Boris Belitsky**

USA

**Christophe Beloin**

France

**Jorge Benach**

USA

**Jean-Marc Berjeaud**

France

**David Bermudes**

USA

**Leticia Bernal-Martínez**

Spain

**Michael Berney**

USA

**Frederic Bertels**

Switzerland

**YZ Bi**

China

**Elena Biagi**

Italy

**Kyle Bibby**

USA

**Michael Bidochka**

Canada

**Elaine Bignell**

UK

**Gursonika Binepal**

Canada

**Emanuele Biondi**

France

**Lucia Birosová**

Slovakia

**Kimberly Bishop-Lilly**

USA

**Debabrata Biswas**

UK

**Jonathan Blackburn**

South Africa

**Dominique Blanc**

Switzerland

**Jorge Blanco**

Spain

**Lucas Blanton**

USA

**JM Blasco**

Spain

**Elisabetta Blasi**

Italy

**Lydia Bogomolnaya**

USA

**Albert Bolhuis**

UK

**Elisa Bona**

Italy

**Rémy Bonnin**

France

**Kajohn Boonrod**

Germany

**Paola Borella**

Italy

**Marco Bottino**

USA

**Ons Bouchami**

Portugal

**Laurent Bouillaut**

USA

**Pascale Bourhy**

France

**Donald Bouyer**

USA

**Edmee Bowles**

The Netherlands

**Sophie Brameyer**

Germany

**Suzanna Brauer**

USA

**Jorge Bresciano**

Spain

**Simon Bridge**

UK

**Thorsten Brinkhoff**

UK

**Andre Brito**

USA

**Edward Broughton**

USA

**Alistair Brown**

UK

**Jonathon Brown**

UK

**Robert Brown**

USA

**Volkmar Brown**

Germany

**Jean-François Brugère**

France

**Brian Brunelle**

USA

**Markus Buchhaupt**

Germany

**Matthias Bureik**

China

**Andreas Burkovski**

Germany

**John Burnett**

USA

**Eric Burrough**

USA

**Filipe Cabreiro**

UK

**Heng Cai**

China

**Alexandra Calteau**

France

**Carlos Camargo**

Brazil

**Floriana Campanile**

Italy

**David Campbell**

USA

**Adrian Canizalez-Roman**

Mexico

**Marcelo Carazzolle**

Brazil

**Barbara Cardazzo**

Italy

**Sherwood Casjens**

USA

**Jim Cassat**

USA

**Marie-Pierre Castanié-Cornet**

France

**Anna Castellazzi**

Italy

**Vincent Cattoir**

France

**Julien Cauchard**

France

**Jean-François Cavin**

France

**Daniela Ceccarelli**

USA

**Marina Cerquetti**

Italy

**Nadia Chacko**

USA

**Yunrong Chai**

USA

**Stephane Chaillou**

France

**Angkana Chaiprasert**

Thailand

**Josephine Chandler**

USA

**In Seop Chang**

South Korea

**Trevor Charles**

Canada

**Keya Chaudhuri**

India

**Esteban Chaves-Olarte**

Costa Rica

**Jiangang Chen**

USA

**Lanming Chen**

China

**Ming-Ju Chen**

Taiwan

**Sheng Chen**

Hong Kong

**Si Chen**

China

**Swaine Chen**

Singapore

**Yanjiong Chen**

China

**Zhilei Chen**

USA

**Huicai Cheng**

China

**Tianyin Cheng**

China

**Luciane Chimetto**

Brazil

**Jeongjoon Choi**

USA

**Youngnim Choi**

South Korea

**Chariya Chomvarina**

Thailand

**Yit Heng Chooi**

Australia

**Rukhsana Chowdhury**

India

**Myron Christodoulides**

UK

**Tinchun Chu**

USA

**Yi-Ping Chuang**

Taiwan

**Takehisa Chuma**

Japan

**Dolores Cid**

Spain

**Maysa Clementino**

Brazil

**Timothy Clough**

New Zealand

**Paul S Cohen**

USA

**Jeff Cole**

UK

**Stéphane Compant**

Austria

**Ian Connerton**

UK

**Fiona Constable**

Australia

**Carmen Contreras**

Mexico

**David Conway**

UK

**Fiona Cooke**

UK

**Geoffrey Coombs**

Australia

**Vaughn Cooper**

USA

**Muriel Cornet**

France

**Adriana Correa**

Colombia

**Antonio Correia**

Portugal

**Marco Cosentino Lagomarsino**

France

**Jacques Covés**

France

**Evan Crawford**

Canada

**ME Casey Crooks**

USA

**Jenny Crowe**

UK

**Christiane Dahl**

Germany

**Paulo Damasco**

Brazil

**Ziyaad Dangor**

South Africa

**Charles Darkoh**

USA

**Bhabatosh Das**

India

**Sujoy Das Gupta**

India

**Gregory Dasch**

USA

**Emmanuelle Dé**

France

**Daniela De Biase**

Italy

**Maria Antonia De Francesco**

Italy

**Tania De Koning-Ward**

Australia

**Gabriel de la Fuente**

Spain

**Rene De Mot**

Belgium

**Jennifer Debruyn**

USA

**Margaret Deighton**

Australia

**Itaru Dekio**

Japan

**Rosa Del Campo**

Spain

**Giovanni Delogu**

Italy

**Plamen Demirev**

USA

**Henk Den Bakker**

USA

**Jonathan Dennis**

Canada

**Marcos Derita**

Argentina

**Abhishek Deshpande**

USA

**Ruud Deurenberg**

The Netherlands

**Kevin Devine**

Ireland

**Rebekah Devinney**

Canada

**Barbara Di Ventura**

Germany

**George Diallingas**

Greece

**Beatriz Eugenia Diez Moreno**

Chile

**George Dimopoulos**

Greece

**Thierry Doan**

France

**Delfina C Dominguez**

USA

**Janet Donaldson**

USA

**Mark Dopson**

Sweden

**Nick Dorrell**

UK

**Sharon Doty**

USA

**Yuetan Dou**

USA

**Andrew Doxey**

Canada

**Klaus Dreisewerd**

Germany

**Kannika Duangmal**

Thailand

**Robert Duda**

USA

**Alain Dufour**

France

**Birgitta Duim**

The Netherlands

**Veeramuthu Duraipandiyan**

India

**Jonathan Dworkin**

USA

**Jaroslaw Dziadek**

Poland

**Markus Egert**

Germany

**Soudeh Ehsani**

Switzerland

**Sigrun Eick**

Switzerland

**Tobias Eisenberg**

Germany

**Wolfgang Eisenreich**

UK

**Johanna Elfenbein**

USA

**Wael S El-Sayed**

Saudi Arabia

**Mohamed Hassan Emara**

Egypt

**Mark Eppinger**

USA

**Romilio Espejo**

Chile

**Ana Esteves**

Portugal

**Paulina Estrada-de los Santos**

Mexico

**Shiuh-Bin Fang**

Taiwan

**Kayode Fashae**

Nigeria

**Denis Faure**

France

**Guido Favia**

Italy

**Luca Federici**

Italy

**Conor Feehily**

Ireland

**Daolun Feng**

China

**Florence Fenollar**

France

**Mithila Ferdous**

The Netherlands

**Chiara Ferrario**

Italy

**David Fewer**

Finland

**Tomas Fiedler**

Germany

**Vale Filipa**

Portugal

**Peter Fineran**

New Zealand

**Luiz Fischer**

Germany

**Nahuel Fittipaldi**

Canada

**Jacqueline Fletcher**

USA

**Ad Fluit**

The Netherlands

**Robin Flynn**

UK

**Gábor Földvári**

Hungary

**Jason Folster**

USA

**Marise Fonseca Dos Santos**

Brazil

**Stephen Forsythe**

UK

**Giovanna Franciosa**

Italy

**Matthew Francis**

Sweden

**Jean-Marie Francois**

France

**Maria Fredriksson-Ahomaa**

Finland

**Christopher French**

USA

**Stephan Fuchs**

Germany

**Masaki Fujita**

Japan

**Kenji Fukui**

Japan

**Vincenzina Fusco**

Italy

**Miklós Füzi**

Hungary

**Attila Gacser**

Hungary

**Aniket Gade**

India

**Lionel Gagnevin**

France

**Juan Carlos Galan**

Spain

**Massimiliano Galdiero**

Italy

**Rodrigo Galhardo**

Brazil

**Antonia Gallo**

Italy

**Giuseppe Gallo**

Italy

**Yunn Gan**

Singapore

**Haichun Gao**

China

**Jin Ming Gao**

China

**Erin Garcia**

USA

**José García**

Spain

**Cristina Garcia-Aljaro**

Spain

**Diego Garcia-Gonzalo**

Spain

**Jose F Garcia-Mazcorro**

Mexico

**Piotr Garstecki**

Poland

**Debora Garzetti**

Germany

**Beilei Ge**

USA

**Otto Geiger**

Mexico

**Shizhong Geng**

China

**Wenqing Geng**

China

**Joan Geoghegan**

Ireland

**Murad Ghanim**

Israel

**Maria Giannouli**

Italy

**Jean-Christophe Giard**

France

**Lindsay Gielda**

USA

**Peter Gilligan**

USA

**Mathieu Gissot**

France

**Philippe Glaser**

France

**Vasiliki Gogou**

Greece

**JP Goldring**

South Africa

**Dasantila Golemi-Kotra**

Canada

**Cassiano Gonçalves-de-Albuquerque**

Brazil

**Angela Gonzalez**

Colombia

**Narjol Gonzalez-Escalona**

USA

**Natalia Gottig**

Argentina

**Sharlene Govender**

South Africa

**Revathi Govind**

USA

**Jayaraman Gowrishankar**

India

**David Grainger**

UK

**Ingrid Green**

UK

**Andrew Greenhill**

Australia

**Gustavo Gregoracci**

Brazil

**Richard Gregory**

USA

**Christopher J Grim**

USA

**Dennis Gross**

USA

**Abhinav Grover**

India

**Sunita Grover**

India

**Kristina Gruden**

Slovenia

**Shao-Bin Gu**

China

**Michael Guarnieri**

USA

**Erwan Gueguen**

France

**Thomas Guillard**

France

**Andrew Gulick**

USA

**Marianne Gunell**

Finland

**Ivan Gunjaca**

Croatia

**Renu Gupta**

India

**Vijai Gupta**

Ireland

**Santiago Gutierrez**

Spain

**Armel Guyonvarch**

France

**Bart Haagmans**

The Netherlands

**Abderrahman Hachani**

UK

**Evelyn Hackl**

Austria

**Stephane Hacquard**

Germany

**Yitzhak Hadar**

Israel

**Maria Hadjifrangiskou**

USA

**Fakhri Haghi**

Iran

**Tsuneo Hakoyama**

Japan

**Michael Hall**

Canada

**Ruth Hall**

Australia

**Jaroslava Halper**

USA

**Larry Halverson**

USA

**Riadh Hammami**

Tunisia

**Brian Hammer**

USA

**Sven Hammerschmidt**

Germany

**Anette M Hammerum**

Denmark

**David Hampson**

Australia

**Chongxu Han**

China

**Lee Han**

Malaysia

**Ruyang Han**

USA

**Sang-Wook Han**

South Korea

**Seung Hyun Han**

South Korea

**Yanping Han**

China

**Anne-Marie Hansen**

USA

**Hilde Hansen**

Norway

**Nicole Hansmeier**

Germany

**Kazuki Harada**

Japan

**Jens Harder**

Germany

**Timm Harder**

Germany

**Gail Hardy**

USA

**Josee Harel**

Canada

**Joe Harrison**

Singapore

**Colin Harwood**

UK

**Fariha Hasan**

Pakistan

**Rumina Hasan**

Pakistan

**CC Hase**

USA

**Tadao Hasegawa**

Japan

**Tamara Haselkorn**

USA

**Lucy Hathaway**

Switzerland

**Bernhard Haubold**

Denmark

**Philippe Hauser**

Switzerland

**Yoichi Hayakawa**

Japan

**Franklin Hays**

USA

**Chunguang He**

China

**Dwayne Hegedus**

Canada

**Rosalee S Hellberg**

USA

**Christopher Hemme**

USA

**Andrew Henderson**

Australia

**Tory Hendry**

USA

**Michael Hensel**

Germany

**Moon Her**

South Korea

**Rosa Hermosa**

Spain

**Josh Herr**

USA

**Enrique Herrero**

Spain

**David Hibbett**

USA

**Paul Hoffman**

USA

**Sven Hoffner**

Sweden

**Martin Holland**

UK

**Emily Hollister**

USA

**Devin B Holman**

Canada

**Mark A Holmes**

UK

**Paul Hooykaas**

The Netherlands

**Fabiana Horn**

Brazil

**Cheng-Hua Huang**

Taiwan

**Qi Huang**

USA

**Olivier Huck**

France

**Stephan Huehn**

Germany

**Alan Huett**

UK

**Gary Huffnagle**

USA

**David Hughes**

Ireland

**Mamie Hui**

Hong Kong

**Sabine Hunke**

Germany

**Martha Hunter**

USA

**Gregory Hurst**

USA

**Darrell Hurt**

USA

**Wilhelmina Huston**

Australia

**Kevin Hybiske**

USA

**Adriana Ianieri**

Italy

**Kobayashi Ichizo**

Japan

**Karunasagar Iddya**

India

**Evgeny Idelevich**

Germany

**Iliyan Iliev**

USA

**Jeongdae Im**

USA

**Robert Imberti**

Italy

**Masayuki Inui**

Japan

**Tobial J Erb**

Germany

**Françoise Jacob-Dubuisson**

UK

**Anne Jamet**

Portugal

**Anuradha Janakiraman**

USA

**Christoph Jans**

Switzerland

**Ruud Jansen**

The Netherlands

**Nico Jehmlich**

Germany

**Freda Jen**

Australia

**Dieter Jendrossek**

Germany

**Byeonghwa Jeon**

Canada

**Kumaraswamy Jeyaram**

India

**Anders Johansson**

Sweden

**Hope Johnson**

USA

**Karyn Johnson**

Australia

**David Joly**

Canada

**Jessica Jones**

USA

**John Jones**

UK

**Malcolm Jones**

Australia

**Gabriela Jorge Da Silva**

Portugal

**Lovleen Tina Joshi**

UK

**Sheryl Justice**

USA

**Vladimir Kaberdin**

Spain

**Tsutomu Kakuda**

Japan

**Marc Karam**

Lebanon

**Enusha Karunasena**

USA

**Hiroaki Kasai**

Japan

**Vera Katalinic-Jankovic**

Croatia

**Sophia Kathariou**

USA

**Jai Kaushik**

India

**Kevin Kavanagh**

Ireland

**Oliver Kayser**

Germany

**Jacqui Keenan**

New Zealand

**Nemat Keyhani**

USA

**Nabil Killiny**

USA

**Kun-Soo Kim**

South Korea

**Lou Kim**

USA

**Kouji Kimura**

Japan

**Marek Kirs**

USA

**Naomi Kobayashi**

Japan

**Gerwald Koehler**

USA

**Michael Koepke**

USA

**Kevin D Kohl**

USA

**Thomas Kohler**

Germany

**Rebekka Kohlmann**

Germany

**OA Koksharova**

Russia

**Irina Kontsevaya**

Russia

**Mirjam Kooistra-Smid**

The Netherlands

**Hnanu Korkeala**

Finland

**Victoria Korolik**

Australia

**Ilias Kounatidis**

UK

**Anne Krachler**

UK

**Mary Krauland**

USA

**Laurent Kremer**

France

**Andreas Kremling**

Germany

**Bastiaan Krom**

The Netherlands

**Alejandra Kruger**

Argentina

**Malgorzata Krzyzowska**

Poland

**Zheng Kuang**

USA

**Sherry Kuchma**

USA

**Mikhail Kudryashev**

Switzerland

**Joosu Kuivanen**

Finland

**Ananda Kulal**

India

**Ayush Kumar**

Canada

**Pradeep Kumar**

Israel

**Tomomi Kuwahara**

Japan

**Pasqualina Laganà**

Italy

**Prem Lakshmanane**

USA

**Rup Lal**

India

**Michael Lalk**

Germany

**Keith Lampel**

USA

**Paolo Landini**

Italy

**Charles Larson**

USA

**Xavier Latour**

France

**Garry Laverty**

UK

**Blair Lawley**

New Zealand

**Hervé Le Moual**

Canada

**Deric Learman**

USA

**David Lebeaux**

France

**François Lebreton**

USA

**Delphine Lechardeur**

France

**Gregor Leckie**

USA

**Bee W Lee**

Singapore

**Dong-Gun Lee**

South Korea

**Hung-Chang Lee**

Taiwan

**John Hwa Lee**

South Korea

**Min-Shi Lee**

Taiwan

**Richard Lee**

USA

**Angelika Lehner**

Switzerland

**Manoel Lemos**

Brazil

**Olivier Lesouhaitier**

France

**Fabien Létisse**

France

**Andre Levesque**

Canada

**Simon Lévesque**

Canada

**Janina Lewis**

USA

**Lisa Lewis**

USA

**Dongmei Li**

USA

**Jiyao Li**

UK

**Zheng-Xi Li**

China

**Haihua Liang**

China

**Li Liang**

USA

**Shuang Liang**

USA

**Yuchieh Liao**

Taiwan

**Carmen Limon**

Spain

**Kristina Lindstrom**

Finland

**Dennis Linton**

UK

**Changhong Liu**

China

**Christine Liu**

USA

**Fei Liu**

China

**Huitao Liu**

USA

**Lingzhi Liu**

USA

**Yuying Liu**

USA

**Zheng-Fei Liu**

China

**Marcial Lizárraga-Partida**

Mexico

**Montserrat Llagostera**

Spain

**Roland Lloubes**

France

**Hsiu-Jung Lo**

Taiwan

**Nathan Lo**

Australia

**Octavio Loera**

Mexico

**Catherine Logue**

USA

**Fernanda Lopes**

Brazil

**Chun Lu**

China

**Yingjie Lu**

USA

**Jay Lucidarme**

UK

**Aneta Luczkiewicz**

Poland

**Carsten Lüder**

Germany

**Jutta Ludwig-Müller**

Germany

**Taina Lundell**

Finland

**Jian Ma**

China

**Michael Mahan**

USA

**Geoffrey Mainda**

UK

**Tim Maisch**

Germany

**Aparna Maiti**

USA

**Caterina Mammina**

Italy

**Francesca Mancianti**

Italy

**Nicasio Mancini**

UK

**Gerardo Manfreda**

Italy

**Jim Manos**

Australia

**Karen Mansfield**

UK

**Angel Manteca**

Spain

**Audelo Naranjo Juan Manuel**

Mexico

**Marina Marcet-Houben**

Spain

**Maria Marco**

USA

**Richard Marconi**

USA

**Imma Margarit**

Italy

**Abelardo Margolles**

Spain

**Michel Marin**

Brazil

**Rumyana Markovska**

Bulgaria

**Sara Marks**

Switzerland

**Gergely Maroti**

Hungary

**Marilis Marques**

Brazil

**Juan Martin**

Spain

**Rebeca Martin Rosique**

France

**Cristina Martinez**

Brazil

**Luis Martínez**

USA

**Manuel Martinez Garcia**

Spain

**Francisco Martínez-Lobo**

Spain

**Esperanza Martinez-Romero**

Mexico

**Sébastien Massier**

France

**Millicent Masters**

UK

**S Matamoros**

The Netherlands

**Ulrike Mathesius**

Australia

**Olga Matos**

Portugal

**Gianluca Matteoli**

Belgium

**Corinne Maurice**

Canada

**Ryan Maves**

USA

**Christelle Mazuet**

France

**Angelo Mazzaglia**

Italy

**John McCulloch**

Brazil

**Martin McGavin**

Canada

**Jodi L McGill**

USA

**Alan McNally**

UK

**Wim Meijer**

Ireland

**Leo Meile**

Switzerland

**Jorge Castro Mejia**

Mexico

**Joseph Meletiadis**

Greece

**Keira Melican**

Sweden

**Joshua Mell**

USA

**Jay Mellies**

USA

**Domenico Meloni**

Italy

**Antonella Mencacci**

Italy

**Marta Mendes**

Portugal

**Dana Ment**

Israel

**Timothy Meredith**

USA

**Y Merrcado-Flores**

Mexico

**JS Metcalf**

USA

**Benjamin Meyer**

Germany

**Arthur Meyers**

USA

**Khan Mi**

USA

**Chikindas Michael**

USA

**Riviere Michel**

France

**Angela Michelato**

Brazil

**Jan Michiels**

Belgium

**Virginie Mick**

France

**Ruth Miller**

Canada

**William Miller**

USA

**Owain Millington**

UK

**Roberto Mioso**

Brazil

**Devesh Misra**

USA

**Jens Moeller**

Switzerland

**Michelle Moffitt**

Australia

**George Moldovan**

USA

**Maelle Molmeret**

France

**Monica Monaco**

Italy

**Vicente Monedero**

Spain

**Marc Monot**

France

**Enrique Monte**

Spain

**Valdirene N Monteiro**

Brazil

**Ji-Won Moon**

USA

**Catrin Moore**

UK

**Amram Mor**

Israel

**Giulia Morace**

Italy

**Yusuke Morimoto**

Japan

**Ignacio Moriyon**

Spain

**Dimitris Mossialos**

Greece

**Daniel Muller**

France

**Frank-Michael C Müller**

Germany

**Jens Munk**

Denmark

**Mariano Jordi Muria Gonzalez**

Australia

**Edith Myers**

USA

**Hideaki Nagamune**

Japan

**Ravinder Nagpal**

Japan

**Laszlo Nagy**

Hungary

**Kshipra Naik**

India

**Christine Najjuka**

Uganda

**K Nakamura**

Japan

**Gerson Nakazato**

Brazil

**Shigetou Namba**

Japan

**Franz Narberhaus**

Germany

**William Nasser**

France

**Nora Navarro Gonzalez**

Canada

**Juana Maria Navarro Llorens**

Spain

**Jason Neal-McKinney**

USA

**Manabu Nemoto**

Japan

**Live Nesse**

Norway

**Catherine Neuwirth**

France

**Ho T Nhan**

Viet Nam

**David Nicolau**

USA

**Kiel Nikolakakis**

USA

**Vladyslav Nikolayevskyy**

UK

**Vandana Dua Niyyar**

USA

**Andreas Nocker**

UK

**Mara Nogueira**

Brazil

**Carl Erik Nord**

Sweden

**Ayman Noreddin**

USA

**Francoise Norel**

France

**Ray Norton**

Australia

**Silvia Cristina Nunez**

Brazil

**Edna Nyangale**

UK

**Joshua Obar**

USA

**David O'Callaghan**

France

**Jun Ogawa**

Japan

**Takao Ohashi**

Japan

**Knut Ohlsen**

Germany

**Elizabeth Ohneck**

USA

**Anil Ojha**

USA

**Takuji Oka**

Japan

**Sezer Okay**

Turkey

**Valéria Oliveira**

Brazil

**John Olsen**

Denmark

**Kien Chai Ong**

Malaysia

**Tareq Osaili**

Jordan

**Colette O'Shaughnessy**

USA

**Jorgelina Ottado**

Argentina

**Hong-Yu Ou**

China

**Gudrun Overesch**

Switzerland

**Joerg Overhage**

Germany

**Kirk Overmyer**

Finland

**Sarah Owens**

USA

**Rabindra Padhy**

India

**Azhahianambi Palavesam**

USA

**Lucia Pallecchi**

Italy

**Juan Carlos Palomino**

Belgium

**João Alencar Pamphile**

Brazil

**Jianyi Pan**

China

**Vijay Pancholi**

USA

**Xiuhua Pang**

China

**Joseph Papaparaskevas**

Greece

**Gyungsoon Park**

South Korea

**Woojun Park**

South Korea

**Athanasios Paschos**

Canada

**Miroslav Patek**

Czech Republic

**Ranjana Pathania**

India

**Luciana Paulino**

Brazil

**Thierry Pedron**

UK

**Tony Pembroke**

Ireland

**Antonio Peña**

Mexico

**Jose Penades**

UK

**Ana-Elena Pérez-Cobas**

Spain

**David Perez-Pascual**

France

**Ljubomir Petricevic**

Austria

**Gregory Phillips**

USA

**Roger Pickup**

UK

**Leland Pierson**

USA

**Laure Plener**

Germany

**Eric Pollitt**

UK

**Stefano Pongolini**

Italy

**Michel-Robert Popoff**

France

**Mohammad Pourshafie**

Iran

**Robert Powers**

USA

**Ram Prasad**

USA

**Richard Preziosi**

UK

**Bernard Prior**

South Africa

**Robert Proctor**

USA

**Edoardo Puglisi**

Italy

**Chris Putnam**

USA

**Huan-Long Qin**

China

**Qiwei Qin**

China

**Sunish Radhakrishnan**

India

**Elias Rahal**

Lebanon

**Marika Rai**

UK

**Tracy Raivio**

Canada

**Murugesan Rajaram**

USA

**Shalini Rajkumar**

India

**Aiyagari Ramesh**

India

**Elsa Ramírez**

Mexico

**Rommel Ramos**

Brazil

**Felix Randow**

UK

**Ravi Ranjan**

USA

**Brian Raphael**

USA

**Rino Rappuoli**

Italy

**Philip Rather**

USA

**J Christian Ray**

USA

**Carolus Reinecke**

South Africa

**Barbara Reinhold-Hurek**

Germany

**Han Remaut**

Belgium

**Fabienne Remize**

France

**Jose Requena**

Spain

**Fredrik Resman**

Sweden

**Diane Retallack**

USA

**Mark Reuter**

UK

**Maria Del Rocio Reyes-Montes**

Mexico

**Hannah Reynolds**

USA

**Jon Marc Rhoads**

USA

**Kelly Rice**

USA

**Peter Richard**

Finland

**S Dean Rider**

USA

**Christian Riedel**

Germany

**Kristian Riesbeck**

Sweden

**German Rivas**

Spain

**Gonzalo Rivera**

USA

**Rafael Rivilla**

Spain

**Michael Robeson**

USA

**Solomon Robinsondavid Jebakumar**

India

**Edson Rocha**

USA

**Tatiana Rochat**

France

**Daniel Rockey**

USA

**Eduardo Rodriguez**

Argentina

**Luiz Roesch**

Brazil

**Judith Rohde**

Germany

**Elina Rohine**

Finland

**Stefano Romano**

Ireland

**Orazio Romeo**

Italy

**Manuel Romero**

UK

**Martin Roop**

USA

**Jason Rosch**

USA

**Baldan Rossella**

Italy

**Ramon Rosselló-Móra**

Spain

**John Rossen**

The Netherlands

**Franca Rossi**

Italy

**Debra Rossouw**

South Africa

**Sophie Roussel**

France

**Utpal Roy**

India

**Fernando Ruiz-Perez**

USA

**Françoise Rul**

France

**Kendra Rumbaugh**

USA

**Andrew Ruys**

Australia

**Sandra Ruzal**

Argentina

**Anna Rymaszewska**

Poland

**Artur Sabat**

The Netherlands

**Sophie Sable**

France

**Michael J Sadowsky**

USA

**Mahdi Saeed**

USA

**Yolanda Sáenz**

Spain

**Jason Sahl**

USA

**Flavia Saia**

Brazil

**Anu Salminen**

Finland

**Gloria Sanchez**

Spain

**Vartul Sangal**

UK

**Maria Santagati**

Italy

**Dam Sao Mai**

Viet Nam

**Sarah Saputo**

USA

**Vishwas Saralaya**

India

**Birinchi Sarma**

India

**Amelia Sarmento**

Portugal

**Reetta Satokari**

Finland

**Dominic Sauvageau**

Canada

**Isabel Scaletsky**

Brazil

**Claudio Scazzocchio**

UK

**Hanno Schäfer**

Germany

**Christina Schäffer**

Austria

**Marjolijn Schalk**

The Netherlands

**Monika Schaubeck**

Germany

**Frieder Schaumburg**

Germany

**Lieke Scheepers**

The Netherlands

**Herb Schellhorn**

Canada

**Richard Scheuermann**

USA

**Ingo Schmitz**

Germany

**Ruth Schmitz-Streit**

Germany

**Dirkjan Schokker**

Germany

**Devinder Sehgal**

India

**Kate Seib**

Australia

**Verena Seidl-Seiboth**

Austria

**Ryan Seipke**

UK

**David Sela**

USA

**Torsten Semmler**

Germany

**Sarah Sengstake**

The Netherlands

**Noha Seoudi**

UK

**Pascale Serror**

France

**Martina Seruga Music**

Croatia

**Alain L Servin**

France

**Rosa Sessa**

Italy

**Migun Shaka**

USA

**Mohammad Reza Shakibaie**

Iran

**Masoomeh Shams-Ghahfarokhi**

Iran

**Nathan Shankar**

USA

**Orin Shanks**

USA

**Mamta Sharma**

India

**Prem Sharma**

India

**Batu Sharma-Kuinkel**

USA

**Lindsey Shaw**

USA

**Rosemary She**

USA

**Wei-Chiang Shen**

Taiwan

**Xiaolin Shen**

China

**Xianming Shi**

China

**Pottathil Shinu**

Saudi Arabia

**Daisuke Shioimi**

Japan

**Brian Shirey**

USA

**Vicky Siarkou**

UK

**Berhanu Sibhat**

Ethiopia

**T Nicolai Siegel**

Germany

**Sanna Sillankorva**

Portugal

**Maria Do Rosario Rodrigues Silva**

Brazil

**Sónia Silva**

Portugal

**Wanderson Silva**

Brazil

**Philippe Simoneau**

France

**Durg Singh**

India

**Suman Singh**

India

**Marcelo Sircili**

Brazil

**Sinosh Skariyachan**

India

**Mikael Skurnik**

Finland

**Pieter Smit**

Sweden

**C Jeffrey Smith**

USA

**Darren Smith**

UK

**Lotte Sogaard-Andersen**

Germany

**Christian Sohlenkamp**

Mexico

**Cristina Solano**

Spain

**Sameh Soliman**

USA

**Avinash Sonawane**

India

**Chunxu Song**

The Netherlands

**Fuping Song**

China

**Yajun Song**

China

**Giuseppe Spano**

Italy

**Barbara Spellerberg**

Germany

**Felipe Sperandio**

Brazil

**Brad Spiller**

UK

**Stanley Spinola**

USA

**Stephen Spiro**

USA

**Andrea Squartini**

Italy

**Abhishek Srivastava**

India

**James Stafford**

Canada

**Juergen Stech**

Germany

**Torsten Sterzenbach**

Germany

**Ann Stevens**

USA

**Scott Stibitz**

USA

**Matt Stoll**

USA

**Douglas Storey**

Canada

**Rhona Stuart**

UK

**David J Studholme**

UK

**Garret Suen**

USA

**Baolin Sun**

China

**Ming Sun**

China

**Meera Sundaram**

USA

**M Sundararaman**

India

**Eric Sundberg**

USA

**Iain Sutcliff**

UK

**Eva Sverremark-Ekström**

Sweden

**Bryan Swingle**

USA

**Wesley Swingley**

USA

**Moriah Szpara**

USA

**Eduardo Taboada**

Canada

**Getachew Tadesse**

Ethiopia

**Kanako Tago**

Japan

**Kapil Tahlan**

Canada

**Eriko Takano**

UK

**Jon Takemoto**

USA

**Rodrigo Taketani**

Brazil

**Ben Tall**

USA

**James Tambong**

Canada

**Michael Tanck**

The Netherlands

**EK Tangni**

Belgium

**Yaser Tarazi**

Jordan

**Aldo Tava**

Italy

**A Tavares**

Brazil

**Elisa Taviani**

Mozambique

**Gianluca Tettamanti**

Italy

**Nikhil Thomas**

Canada

**Thomas Thurnheer**

Switzerland

**Uwe Tietge**

UK

**Stephen Todryk**

UK

**Masanori Tohno**

Japan

**Andrew Tolonen**

France

**Maria Tomas**

Spain

**Jan Tommassen**

The Netherlands

**Liam Toner**

Australia

**Bertrand Toussaint**

France

**Chanwit Tribuddharat**

Thailand

**Susannah Tringe**

USA

**Stephen Tristram**

Australia

**Athanassios Tsakris**

Greece

**Francesca Turroni**

Italy

**Toshiki Uchiumi**

Japan

**Takashi Uebanso**

Japan

**Shah Ali Ul Qader**

Pakistan

**Glen Ulett**

Australia

**María Teresa Ulloa Flores**

Chile

**RS Upadhyay**

India

**Constantin Urban**

Sweden

**María Sofía Urbieta**

Argentina

**Yutaka Uyeno**

Japan

**Anni Vainio**

Finland

**Mariadhas Valan Arasu**

Saudi Arabia

**Susanna Valanne**

Finland

**Sylvia Valdezate**

Spain

**Hannamari Välimaa**

Finland

**Alje Van Dam**

The Netherlands

**Mark Van Der Linden**

Germany

**Jan-Roelof van der Meer**

Switzerland

**Henny Van der Mei**

The Netherlands

**Menno van der Voort**

The Netherlands

**Jan Maarten van Dijl**

The Netherlands

**Paul van Heusden**

The Netherlands

**Noortje van Maarseveen**

The Netherlands

**Willem van Schaik**

The Netherlands

**Alessandro M Varani**

Brazil

**Martha Vaughan**

USA

**Willem Veening**

The Netherlands

**Mohanan Veettil**

USA

**Tony Velkov**

Australia

**Alida Veloo**

The Netherlands

**Lakshmi Vemu**

India

**K Vengadesan**

India

**André Verméglio**

France

**Alex Vieira Chaves**

Australia

**Shripal Vijayvargiya**

India

**Julio Villena**

Argentina

**Miguel Vinas**

Spain

**Delphine Vincent**

Australia

**Joseph Vinetz**

USA

**Karen Visick**

USA

**Miguel Viveiros**

Portugal

**Anastasia Vlasova**

USA

**Jiri Volf**

Czech Republic

**Ingemar Von Ossowski**

Finland

**Roel Vonk**

The Netherlands

**Frank Vriesekoop**

UK

**Sun Nyunt Wai**

Sweden

**Pegine Walrad**

UK

**Guoliang Wang**

USA

**Jianchuan Wang**

USA

**Jinglin Wang**

China

**Jingxue Wang**

China

**Jingyu Wang**

USA

**Liping Wang**

China

**Shaohua Wang**

China

**Shihua Wang**

China

**Xuan Wang**

USA

**Zhang Wang**

USA

**Zheng Wang**

USA

**Trudy Wassenaar**

Germany

**Koichi Watanabe**

Japan

**Pierre Wattiau**

Belgium

**Grzegorz Wegrzyn**

Poland

**Silja Wessler**

Austria

**Christopher West**

USA

**Edze Westra**

USA

**Robert Whiley**

UK

**Andrew Whitelaw**

South Africa

**Anne Willems**

Belgium

**Flavia Winck**

Brazil

**Klaus Winzer**

UK

**Wolfgang Witte**

Germany

**Alan Wolfe**

USA

**Charles Woloshuk**

USA

**Nicole Wolter**

South Africa

**HC Wong**

Taiwan

**Han Min Woo**

South Korea

**Nancy Woychik**

USA

**Jian Wu**

USA

**Qinglong Wu**

China

**Wai Lan Wu**

Hong Kong

**Jie Xiao**

USA

**Jingfa Xiao**

China

**Qiaobin Xiao**

USA

**Howard Xu**

USA

**Jiannong Xu**

USA

**Meiying Xu**

China

**Manisha Yadav**

India

**Mamoru Yamada**

Japan

**Yoshio Yamaoka**

USA

**Masahito Yamazaki**

Japan

**Jun Yan**

USA

**Ence Yang**

USA

**Guang Yang**

China

**Jun-Yi Yang**

Taiwan

**Liang Yang**

Singapore

**Qianru Yang**

USA

**Rongchang Yang**

Australia

**Shihui Yang**

USA

**Qiuming Yao**

USA

**Aymen S Yassin**

Egypt

**Gong-Yin Ye**

China

**Iris Yehidia**

Israel

**Qingqin Yin**

China

**Sukhwan Yoon**

South Korea

**Chris Yost**

Canada

**Huimin Yu**

China

**Yuhe Yu**

China

**Zechun Yuan**

Canada

**Nicola Zamboni**

Switzerland

**Maria Mercedes Zambrano**

Colombia

**Raffaele Zarrilli**

Italy

**Aleksandra Zasanda**

Poland

**Alexandre Zavascki**

Brazil

**Einat Zchori-Fein**

Israel

**Rintze Zelle**

USA

**Haili Zhang**

USA

**Huiwen Zhang**

China

**Lu Zhang**

USA

**Qijing Zhang**

USA

**Ying Zhang**

USA

**Jinlei Zhao**

USA

**Yong Zhao**

China

**Youfu Zhao**

USA

**Dongsheng Zhou**

China

**Hongwei Zhou**

China

**Wei-Yun Zhu**

China

**Wei-Qin Zhuang**

USA

**Ayrat Ziganshin**

Russia

**Dezemon Zingué**

Burkina Faso

**Christian Zmasek**

USA

**Sadri Znaidi**

France

**Aldert Zomer**

The Netherlands

**Huasong Zou**

China

**Roland Züst**

Singapore

